# YB-1 and MTA1 protein levels and not DNA or mRNA alterations predict for prostate cancer recurrence

**DOI:** 10.18632/oncotarget.3477

**Published:** 2015-03-03

**Authors:** Christine Moore Sheridan, Tristan R. Grogan, Hao G. Nguyen, Colette Galet, Matthew B. Rettig, Andrew C. Hsieh, Davide Ruggero

**Affiliations:** ^1^ Department of Urology, University of California, San Francisco, CA, USA; ^2^ Statistic Core, David Geffen School of Medicine, University of California, Los Angeles, CA, USA; ^3^ Department of Medicine, VA Greater Los Angeles Healthcare System-West Los Angeles, CA, USA; ^4^ Department of Urology, School of Medicine, University of California, Los Angeles, CA, USA; ^5^ Department of Medicine, Division of Hematology/Oncology, University of California, San Francisco, CA, USA; ^6^ Present address: Division of Human Biology, Fred Hutchinson Cancer Research Center, Seattle, WA, USA

**Keywords:** translation control, prostate cancer, PSA recurrence, biomarker, YB-1

## Abstract

Attempts to identify biomarkers to detect prostate tumorigenesis, and thus minimize prostate cancer progression and inform treatment decisions have primarily focused on alterations at the DNA and mRNA levels, ignoring alterations at the level of protein synthesis control. We have previously shown that the PI3K-AKT-mTOR pathway, frequently deregulated in prostate cancer, specifically induces the synthesis of proteins that contribute to metastasis, most notably YB-1 and MTA1, without altering mRNA levels thereby demonstrating the importance of translation control in driving the expression of these genes in cancer. Here, we analyze genomic sequencing and mRNA expression databases, as well as protein expression employing an annotated tissue microarray generated from 332 prostate cancer patients with 15 years of clinical follow-up to determine the combined prognostic capability of YB-1 and MTA1 alterations in forecasting prostate cancer outcomes. Remarkably, protein abundance, but not genomic or transcriptional alterations of YB-1 and MTA1, is predictive of disease recurrence, exhibiting a dose-dependent effect on time to PSA recurrence, an indicator of tumor relapse. Moreover, high protein levels of YB-1 and MTA1 are associated with a 3-fold increased risk for requiring future hormone therapy or radiation therapy. Importantly, YB-1 and MTA1 protein levels significantly increase the predictive capacity of a clinical model for prostate cancer recurrence. These findings demonstrate that protein abundance of YB-1 and MTA1, irrespective of DNA or mRNA status, can predict for prostate cancer relapse and uncover a vast underappreciated repository of biomarkers regulated at the level of protein expression.

## INTRODUCTION

Prostate cancer is the second leading cause of cancer-related deaths among American men, with an estimated 27,540 deaths in 2015. Furthermore, metastatic prostate cancer is currently incurable. Many efforts have been made to identify clinical variables or biomarkers to predict for disease recurrence in post-surgical or radiation therapy patients. In particular, contemporary practices rely on pathologic staging, Gleason score, disease volume, surgical margin status, and pre-operative prostate specific antigen (PSA) levels and kinetics to predict for the likelihood of disease relapse after definitive care [1-3]. However, many patients with low probability of relapse based on these factors still develop PSA recurrence, which is typically the first indicator of disease relapse in prostate cancer patients after surgery and ultimately leads to lethal metastases. Thus, a critical bottleneck in prostate cancer patient care is the ineffective criteria we currently use to identify patients early on in the disease process who may eventually recur. The identification of biomarkers that can predict for future disease states is critical to our ability to decrease patient morbidity and mortality associated with prostate cancer.

To this end, genomic and transcription-based biomarkers have been widely utilized to predict outcomes in patients with prostate cancer, but have been of limited clinical utility. One important factor underlying the inadequacy of these predictors is that alterations at the DNA and RNA levels are frequently not reflected at the level of protein expression [4], which is ultimately required to enable specific cellular functions that drive tumor initiation and progression. Regulation of protein synthesis is a critical step of gene expression that dictates the milieu of proteins that ultimately make up a cancer cell. Moreover, there is mounting evidence that deregulation of the translation machinery is necessary for prostate cancer pathogenesis [5, 6]. This is mediated in part through oncogenic signaling networks such as the PI3K-AKT-mTOR pathway, which is commonly deregulated in human prostate cancer [7], and has been shown to direct the translation of distinct groups of mRNAs, modulating their protein expression without altering their transcript levels [5, 8]. Importantly, many of these translationally regulated mRNAs direct vital cellular functions necessary for cancer progression. Notably, a group of these PI3K-AKT-mTOR translational targets including the Y-box binding protein-1 (YB-1) and the chromatin remodeling protein metastasis associated-1 (MTA1) can endow prostate epithelial cells with invasive potential and are necessary to maintain the invasive qualities of prostate adenocarcinoma [5]. However, it is not known whether the resulting protein levels of translationally controlled mRNAs such as YB-1 and MTA1 can together predict prostate cancer patient outcomes.

In order to comprehensively determine the step of gene expression in which YB-1 and MTA1 are deregulated in prostate cancer patients, we analyzed publically available genomic and transcript databases, and conducted quantitative immunofluorescence analysis of YB-1 and MTA1 protein levels across a large annotated human prostate cancer tissue microarray with over 15 years of clinical follow-up (Table 1). We determine that YB-1 and MTA1 do not frequently exhibit alterations at the DNA or RNA levels. Instead, our findings indicate a striking correlation between high protein levels of YB-1 and MTA1 with PSA recurrence, which is the first indicator of prostate cancer progression after definitive localized therapy. In line with these findings, patients with high protein levels of YB-1 and MTA1 are three times as likely to require future interventions such as androgen deprivation therapy or radiation therapy after radical prostatectomy. Moreover, the addition of YB-1 and MTA1 biomarkers to commonly used clinical factors including Gleason score, PSA level, seminal vesicle invasion, extracapsular extension, and positive surgical margins significantly increases the predictive potential of a clinical nomogram.

**Table 1:** West Los Angeles Veterans Affairs patient characteristics Patient characteristics of 332 men who underwent radical prostatectomy between 1991 and 2003 at the West Los Angeles Veteran's Administration Medical Center (WLA VA).

	Patients (n = 332)	
Variable	No.	(%)
**Age**		
<65	194	58.4
≥65	138	41.6
**PSA**		
≤4	52	15.7
5-10	167	50.3
>10	113	34
**BMI**		
<25	80	24.1
25-30	144	43.4
≥30	86	25.9
unknown	22	6.6
**Gleason Score**
≤6	179	53.9
7	134	40.4
≥8	19	5.7
**Pathologic Stage**		
T2	273	82.2
T3	46	13.9
T4	13	3.9
**Recurrence Status**		
+	129	38.9
-	200	60.2
unknown	3	0.9
**Margin Status**		
+	144	43.4
-	186	56.0
unknown	2	0.6
**Node Status**		
+	6	1.8
-	280	84.3
unknown	46	13.9
**Extracapsular Extension**		
+	36	10.8
-	295	88.9
unknown	1	0.3
**Seminal Vesicle Invasion**		
+	35	10.5
-	295	88.9
unknown	2	0.6

## RESULTS

### YB-1 and MTA1 are not altered at the DNA and RNA levels in human prostate cancer

We and others previously identified Y-box binding protein 1 (YB-1) and metastasis associated-1 (MTA1) as proteins regulated at the translation, and not mRNA transcription level downstream of the PI3K-AKT-mTOR signaling pathway, which is frequently deregulated in human prostate cancer [5, 7]. YB-1 is an RNA/DNA binding protein with pleiotropic functions including the ability to regulate the post-transcriptional expression of a network of pro-invasion mRNAs [9]. MTA1 is a chromatin remodeling protein, which has been implicated in cancer metastasis [10, 11]. Multiple groups have demonstrated that RNAi of YB-1 and MTA1 *in vitro* leads to a significant decrease in the invasive capacity of cancer cells [5, 9, 12]. However, very little is known about how YB-1 and MTA1 are together deregulated in human prostate cancer tissues. In particular, it is unknown if YB-1 and MTA1 are altered at the genomic or transcriptional levels. We investigated the frequency of mutations or amplifications of these genes at the DNA level utilizing publicly available datasets of sequenced human prostate cancer tissue specimens (http://www.cbioportal.org/public-portal/, Taylor et al. Cancer Cell 2010 [7], Barbieri et al. Nature Genetics 2011 [13], and Grasso et al. Nature 2012 [14] datasets). No mutations or amplifications in human prostate cancer were observed. To determine in an unbiased manner if YB-1 or MTA1 mRNA levels are different between normal prostate tissue and prostate cancer, we analyzed 15 human prostate cancer mRNA datasets published between 2001 and 2012 (Table 2), which included 1016 unique patient samples. Importantly, the majority of the datasets analyzed used tissues that were highly enriched for cancer cells with minimal stromal contamination (Supplementary Material 1). 13 out of 14 datasets showed no statistically significant difference in YB-1 mRNA expression levels (Table 2). For MTA1, 12 out of 15 datasets exhibited no significant difference between normal prostate tissue and prostate cancer (Table 2). Thus, in the majority of prostate cancer mRNA datasets analyzed, no difference in YB-1 and MTA1 mRNA expression was observed. Based on these findings, DNA or RNA alterations of YB-1 and MTA1 cannot distinguish between normal prostate and prostate cancer tissues.

**Table 2:** YB-1 and MTA1 mRNA levels exhibit very little variation between normal and prostate cancer tissues across 15 published human prostate cancer studies Analysis of the fold change of YB-1 and MTA1 transcript levels in normal prostate tissue compared to prostate cancer tissues using the curated Oncomine database (n = 1016 normal and prostate cancer specimens). Red = statistically significant difference between normal versus cancer. n/a = probe not present in dataset. n.s. = not statistically significant.

Study	n	YB-1		MTA1	
		Fold change	P-value	Fold change	P-value
Arredouani et al. CCR 2009	21	1.064	0.318	1.121	0.056
Grasso et al. Nature 2012	122	−1.045	0.758	1.136	0.244
Holzbeierlein et al. Am J Path 2004	54	−1.044	0.614	1.057	0.415
LaTulippe et al. Cancer Research 2002	35	−1.62	0.828	1.064	0.364
Liu et al. Cancer Research 2006	57	1.021	0.378	1.076	0.171
Luo et al. Mol Carcinog 2002	30	1.06	0.338	1.217	0.082
Magee et al. Cancer Research 2001	15	1.397	0.986	2.474	0.011
Singh et al. Cancer Cell 2002	102	1.403	0.007	1.051	0.379
Taylor et al Cancer Cell 2010	185	1.012	0.378	−1.021	0.791
Tomlins et al. Nature Genetics 2007	101	n/a	n/a	−1.167	0.685
Vanaja et al. Cancer Research 2003	40	1.21	0.647	1.168	0.119
Verambally et al. Cancer Cell 2005	19	−1.133	0.830	1.014	0.409
Wallace et al. Cancer Research 2008	89	−1.097	0.608	1.702	2.66E-05
Welsh et al. Cancer Research 2001	34	1.066	0.260	1.696	0.207
Yu et al. JCO 2004	112	−1.121	0.998	1.305	0.002
**n.s. in study/total studies**		**13/14**		**12/15**	

### YB-1 and MTA1 protein levels increase during prostate cancer progression

**Figure 1:**
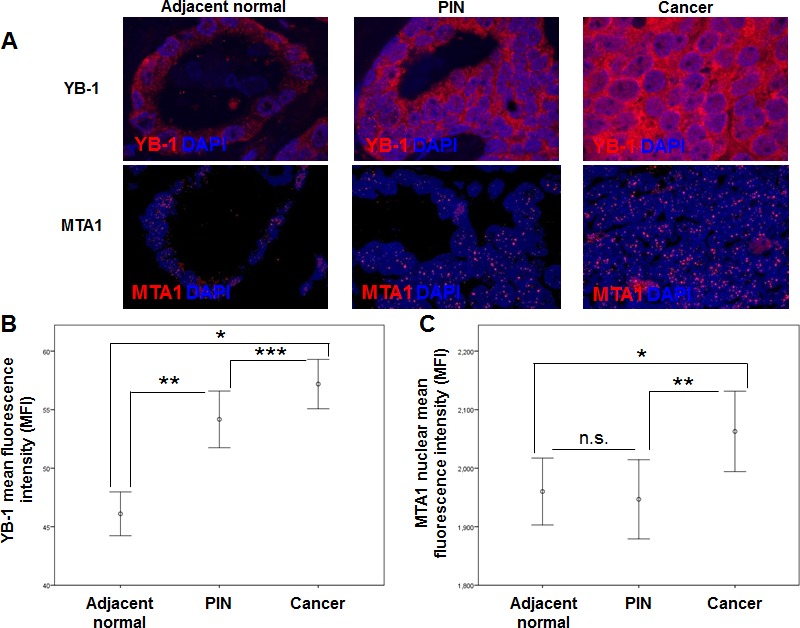
YB-1 and MTA1 protein levels in adjacent normal, prostatic intraepithelial neoplasia (PIN), and cancer samples from WLA VA patient cohort (A) Representative images of adjacent normal, PIN, and prostate cancer tissues stained for YB-1 or MTA1. (B) Mean fluorescence intensity (MFI) of YB-1 in adjacent normal, PIN, and prostate cancer tissues (* P < 0.001, ** P < 0.001, *** P = 0.014, error bars = 95% confidence interval) (C) Mean fluorescence intensity (MFI) of MTA1 in adjacent normal, PIN, and prostate cancer tissues (* P = 0.002, ** P = 0.008, n.s. = not statistically significant, error bars = 95% confidence interval).

Given that YB-1 and MTA1 are translationally regulated mRNAs [5], we hypothesized that differences could be observed at the protein level between normal, pre-invasive prostatic intraepithelial neoplasia (PIN), and prostate cancer tissues despite the lack of alterations at the DNA and mRNA levels. To this end, tissue microarrays were generated using adjacent normal tissue, PIN, and prostatic adenocarcinoma gathered from patients who underwent radical prostatectomy between 1991-2003 at the West Los Angeles Veteran's Affair Medical Center (Table 1). These arrays were stained with YB-1 and MTA1-specific antibodies, imaged using semi-automated high-throughput platforms, and quantified in an unbiased manner. By utilizing this quantitative immunofluorescence technique, we were able to measure the entire spectrum of YB-1 and MTA1 protein expression, from minimally detectable to high levels within each tissue sample. The staining pattern of YB-1 was predominantly cytoplasmic in adjacent normal prostate tissue, PIN, and cancer, which is consistent with its function in regulating the translation of distinct mRNA networks (Figure 1A) [9]. YB-1 protein levels increased in a step-wise manner from normal prostate epithelium to cancer (Figures 1A, 1B, Supplementary Material 2) suggesting a very early requirement for YB-1 in prostate tumorigenesis. In contrast, MTA1 was predominantly nuclear in all three types of tissue (Figure 1A), consistent with its function as a dynamic regulator of chromatin structure [11]. Mean MTA1 levels were nearly equivalent between adjacent normal tissue and PIN, however, PIN exhibited a greater range of MTA1 expression compared to adjacent normal prostate epithelium (Figures 1A, 1C, Supplementary Material 2). In cancer, MTA1 levels are significantly upregulated compared to adjacent normal prostate tissue and PIN (Figures 1A, 1C). Together, this analysis reveals significant increases in YB-1 and MTA1 protein levels throughout prostate cancer pathogenesis, which were not observed at the DNA or RNA levels. Increases in protein synthesis rates likely account for the increase of YB-1 and MTA1 protein levels observed and are consistent with the observation that they are translationally regulated mRNAs [5]. Thus, translationally regulated mRNAs such as YB-1 and MTA1 may serve as biomarkers whose expression trend with the natural history of prostate cancer development and progression.

### Clinical factors do not correlate with YB-1 and MTA1 protein levels

Next, we sought to determine if there was a correlation between YB-1 and MTA1 protein levels and clinical factors historically associated with prostate cancer progression. Spearman's correlation analyses were completed comparing pre-operative PSA levels, Gleason score, presence of seminal vesicle invasion (SVI), presence of extra-capsular extension (ECE), and margin status with YB-1 and MTA1 mean fluorescence intensities. No significant correlation was found in YB-1 and MTA1 expression levels in adjacent normal prostate, PIN, or prostate cancer with any of the clinical factors examined (Table 3). Importantly, these findings highlight that protein levels of YB-1 and MTA1 do not positively correlate with metrics associated with aggressive prostate cancer, but instead raise the possibility that these biomarkers may be independent predictors of disease outcomes in prostate cancer patients undergoing localized therapy.

**Table 3:** Spearman's correlation analysis reveals minimal association between clinical factors and YB-1 or MTA1 protein levels Spearman's correlation analysis between YB-1 and MTA1 protein levels and clinical factors including PSA levels, Gleason score, seminal vesicle invasion (SVI), extracapsular extension (ECE), and surgical margin status. P-values in parentheses.

	YB-1			MTA1		
Clinical Factor	Adjacent normal	PIN	Cacer	Adjacent normal	PIN	Cancer
PSA	−0.014 (0.808)	0.044 (0.52)	0.045 (0.419)	0.012 (0.832)	−0.061 (0.379)	−0.005 (0.923)
Gleason score	0.019 (0.73)	0.025 (0.723)	0.019 (0.729)	0.071 (0.203)	−0.057 (0.408)	0.082 (0.139)
SVI	−0.004 (0.942)	0.068 (0.326)	0.045 (0.416)	−0.037 (0.511)	−0.139 (0.046)	−0.08 (0.151)
ECE	0.015 (0.786)	0.07 (0.31)	0.069 (0.213)	−0.038 (0.499)	−0.063 (0.366)	−0.078 (0.16)
Margin status	0.015 (0.794)	0.002 (0.982)	0.024 (0.672)	−0.073 (0.19)	−0.068 (0.325)	−0.069 (0.211)

### YB-1 and MTA1 protein levels in PIN independently predict for PSA recurrence

**Figure 2:**
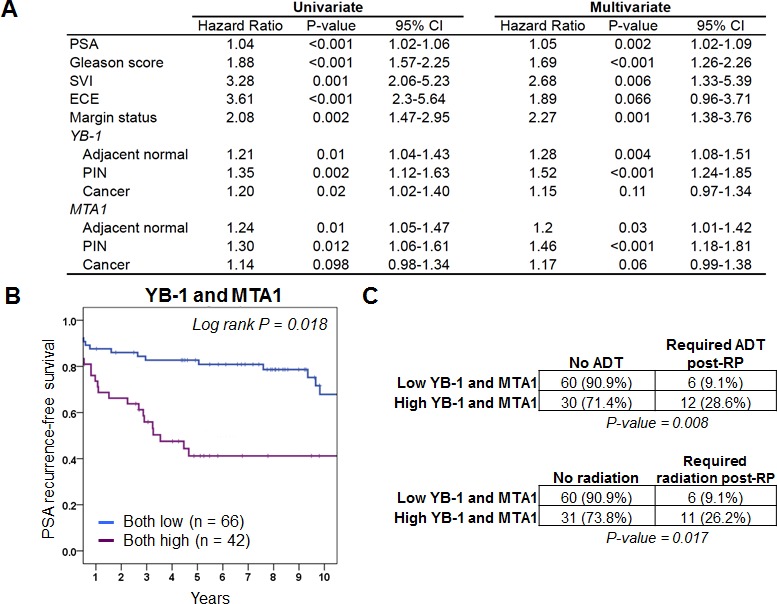
YB-1 and MTA1 protein levels are predictors of PSA recurrence-free survival and future need for androgen deprivation therapy or radiation therapy (A) Univariate and multivariate analysis of pre- and postoperative features for the prediction of PSA progression in 332 patients treated with radical prostatectomy for clinically localized prostate cancer. (B) Kaplan-Meier analysis demonstrates that high protein expression levels of YB-1 and MTA1 within prostatic intraepithelial neoplasia lesions are associated with worse PSA recurrence-free survival. (C) Total number and percentage of patients who required androgen deprivation therapy or radiation therapy post-prostatectomy (post-RP) based on high or low protein levels of YB-1 and MTA1 (Chi-squared test).

We next determined the independent predictive value of YB-1 and MTA1 levels on the clinically important outcome of PSA recurrence, which is typically the first indicator of disease relapse in prostate cancer patients after surgery. As expected, pathologic features such as pre-operative PSA levels, Gleason score, presence of ECE, presence of SVI, and positive surgical margins were all significant predictors of PSA recurrence by both univariate and multivariate analyses (Figure 2A). Strikingly, a univariate analysis revealed a significant risk for PSA recurrence in patients whose adjacent normal, PIN, and prostate cancer tissues expressed high YB-1 protein levels (adjacent normal: HR 1.21, 95% CI 1.04-1.43, P = 0.01; PIN: HR 1.35, 95% CI 1.12-1.63, P = 0.002; cancer: HR 1.20, 95% CI 1.02-1.40, P = 0.02) (Figure 2A). For MTA1, the univariate analysis showed an increased risk for PSA recurrence associated with high protein levels in adjacent normal and PIN, but not prostate cancer tissues (adjacent normal: HR 1.24, 95% CI 1.05-1.47, P = 0.01; PIN: HR 1.30, 95% CI 1.06-1.61, P = 0.012; cancer: HR 1.14, 95% CI 0.98-1.34, P = 0.098) (Figure 2A). The positive association between high YB-1 and MTA1 protein levels in adjacent normal tissues and PSA-recurrence raises the possibility that non-cell autonomous effects elicited by the surrounding microenvironment may also influence the expression of these proteins. Interestingly, these findings also demonstrate that YB-1 and MTA1 protein levels are most predictive for PSA recurrence in a particular tissue type. For example, out of all the tissue types analyzed including adjacent normal, PIN, and prostate cancer, high protein levels of YB-1 and MTA1 in PIN lesions were the most significantly associated with an increased risk for PSA recurrence. The potential predictive capacity of YB-1 and MTA1 protein levels as biomarkers in PIN for PSA recurrence is further supported by a subsequent multivariate analysis, which unlike the univariate analysis, controls for clinical and pathological factors. In particular, increased levels of YB-1 and MTA1 in the PIN samples correlated with the highest risk for PSA recurrence following radical prostatectomy, as compared with YB-1 and MTA1 protein levels in adjacent normal and prostate cancer tissues (PIN: HR = 1.52, 95% CI 1.24-1.85, P < 0.001 and HR 1.46, 95% CI 1.18-1.81, P < 0.001, respectively; adjacent normal: HR 1.28, 95% CI 1.08-1.51, P = 0.004 and HR 1.2, 95% CI 1.01-1.42, P = 0.03, respectively; cancer: HR 1.15, 95% CI 0.97-1.34, P = 0.11 and HR 1.17, 95% CI 0.99-1.38, P = 0.06, respectively) (Figure 2A). Thus, protein levels of YB-1 and MTA1 in PIN lesions from radical prostatectomy tissue samples are the most predictive for PSA recurrence compared to other tissue types. Moreover, these findings raise the intriguing possibility that acquisition of metastatic potential is an early event in prostate cancer development.

### High YB-1 and MTA1 protein levels in PIN are associated with future disease behavior including PSA recurrence and the eventual need for interventions including androgen deprivation therapy and radiation therapy

In order to determine the clinical relevance of high protein expression levels of YB-1 and MTA1 in PIN on PSA recurrence-free survival, Kaplan-Meier analysis was performed. The 5-year PSA recurrence-free survival rates were 46% and 39%, respectively, in patients expressing the highest levels of YB-1 or MTA1 (4^th^ quartile) compared to 79% and 70%, respectively, for the lowest expressers (1^st^ quartile) (Supplementary Materials 3 and 4). Log-rank test showed that the PSA recurrence rate differed significantly between the patients with high levels of YB-1 and MTA1 compared to those with low levels (P = 0.018 and 0.031 respectively) (Supplementary Materials 3 and 4). These findings demonstrate that YB-1 and MTA1 protein levels in PIN lesions can independently serve as prognostic markers for PSA recurrence. However, it is unknown if the two biomarkers can together predict for future disease behavior. To this end, we determined if patients with elevated levels of both YB-1 and MTA1 were more likely to develop PSA recurrence compared to those with low levels of both. The 5-year PSA recurrence-free survival rate was 83% and 42%, respectively, in patients expressing the lowest levels of both YB-1 and MTA1 compared to high expressers (Figure 2B). Thus, based on YB-1 and MTA1 protein levels alone, post-radical prostatectomy patients can be stratified into low or high likelihood of PSA relapse. In addition to PSA recurrence, which is the first indication of prostate cancer progression, we next determined if the protein levels of YB-1 and MTA1 correlate with clinically relevant end points beyond changes in the PSA level including future need for androgen deprivation therapy or radiation therapy. Strikingly, patients with high YB-1 and MTA1 protein levels were three times as likely to require hormone therapy or radiation therapy after a radical prostatectomy (Figure 2C). As such, protein levels of both YB-1 and MTA1 predict for clinically important factors including the need for additional future therapy.

### Combining YB-1 and MTA1 protein levels with clinical factors improves the ability to predict for PSA recurrence

**Figure 3:**
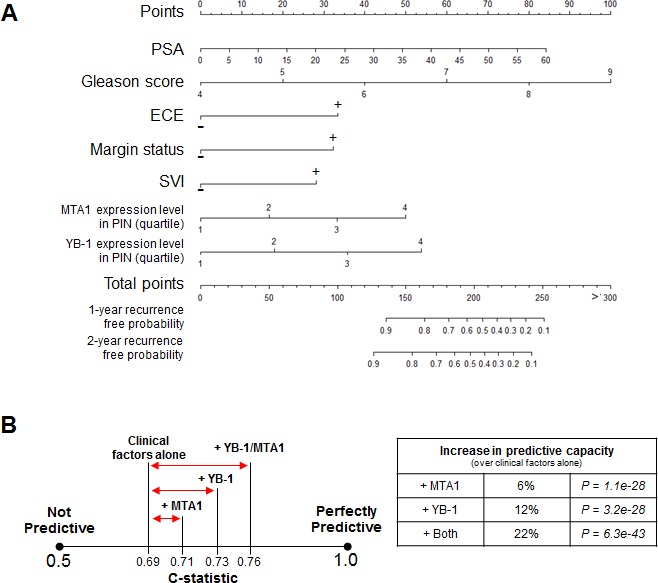
The addition of the MTA1 and YB-1 biomarkers to clinical factors including the Gleason score can improve upon the predictive capacity of a clinical nomogram to forecast PSA progression after radical prostatectomy (A) Nomogram to predict 1-year and 2-year recurrence free probability. (B) C-statistics demonstrating the predictive capacity of the nomogram with clinical factors alone, clinical factors + MTA1, clinical factors + YB-1, and clinical factors + MTA1 + YB-1.

Next, using the clinical data from the 332 patient tissue microarray database, we generated a nomogram composed of Gleason score, PSA level, extracapsular extension, surgical margin status, and seminal vesicle invasion for 1- and 2-year PSA recurrence-free probability. Using this tool, we asked if the addition of the YB-1 and MTA1 biomarkers could increase the predictive capacity of a clinically based nomogram. We evaluated the prognostic ability of the model by calculating the C-statistic, which ranges from 0.5 (not predictive) to 1 (perfectly predictive). The clinical nomogram alone had a C-statistic of 0.69 (P = 2.7e-29) (Figures 3A and 3B). Adding MTA1 or YB-1 protein levels individually to the clinical nomogram increased the C-statistic to 0.71 and 0.73, which represents a 6% (P = 1.1e-28) or 12% (P = 3.2e-28) increase in predictive capacity of the nomogram, respectively (Figure 3B). Remarkably, by combining both biomarkers in the model, the C-statistic rose to 0.76, which is a 22% (P = 6.3e-43) increase in the predictive capacity of the nomogram compared to using clinical factors alone, including the Gleason score (Figure 3B). Thus, a combinatorial approach, which includes clinical factors and translationally regulated biomarkers such as YB-1 and MTA1, represents a potentially powerful method to predict for future prostate cancer behavior after a prostatectomy.

## DISCUSSION

Defining signatures that can predict whether prostate cancer patients have indolent versus aggressive disease is of critical importance to informing treatment interventions and decreasing morbidity and mortality. Previous efforts to identify genomic and transcriptional gene signatures that are clinically useful in prostate cancer have been met with limited success. This is likely due to the inherent discordance between alterations at the genomic and transcriptional levels and the milieu of proteins that are ultimately produced [4]. Importantly, the regulation of protein synthesis is emerging as a vital mechanism to direct cancer cell behavior. A growing body of evidence has demonstrated that specific alterations in the activity of the translation machinery driven by oncogenic signaling pathways direct tumor initiation and progression in prostate cancer through the aberrant translation of distinct mRNA networks, which include YB-1 and MTA1 [5]. However, it is unknown whether translationally regulated mRNA signatures might constitute biomarkers with clinical utility. To this end, we have demonstrated that a previously identified pair of translationally regulated mRNAs encoding for YB-1 and MTA1 [5] are independent predictors for future disease behavior. In particular, high protein levels of YB-1 and MTA1 within PIN lesions are significantly associated with a shorter time to PSA relapse as well as a three fold increased risk for requiring future androgen deprivation therapy or radiation therapy (Figures 2B and 2C). Importantly, these biomarkers also increase the predictive capacity of a clinical nomogram, which includes the Gleason score, to forecast PSA progression post-prostatectomy (Figure 3). It is important to note that YB-1 and MTA1 proteins would not have been identified as potential biomarkers by the analysis of their gene locus or RNA expression (Table 2). As such, our new findings highlight the importance of post-transcriptional control as a means to identify potential novel biomarkers in cancer.

An important question that arises from this study is why elevated levels of YB-1 and MTA1 are most predictive for PSA recurrence in PIN lesions as opposed to cancer lesions? Interestingly, in a mouse model of prostate cancer driven by PI3K-AKT-mTOR hyperactivity [15], elevated protein levels of YB-1 and MTA1 were observed early in disease pathogenesis within PIN lesions prior to the development of invasion and metastasis [5]. Thus, one possibility is that pre-invasive neoplastic epithelial cells may already activate programs that direct future aggressive cancer cell behavior such as metastasis. Importantly, YB-1 and MTA1 have been shown to endow immortalized epithelial cells with invasive properties and are necessary to maintain the invasive potential of prostate cancer cells [5]. As such, high protein levels of YB-1 and MTA1 in PIN may predispose patients to a more aggressive prostate cancer when it develops, thereby increasing the risk for PSA recurrence or the need for future therapy after a prostatectomy. Indeed, these findings are in line with the recent discovery that the molecular characteristics of PIN can predict for future disease development [16]. As such, the identification of YB-1 and MTA1 as predictive biomarkers represents a potentially important advancement, which may allow clinicians to prognosticate the aggressive or indolent nature of a prostate adenocarcinoma that arises within a patient.

These results highlight two possibilities for the application of YB-1 and MTA1 protein levels as biomarkers in human cancers. First, YB-1 and MTA1 levels can be used to risk-stratify prostate cancer patients to distinguish those who may benefit from immediate adjuvant therapy such as radiation after surgery. Second, multiple therapeutics that target the translation machinery are currently in clinical development. Specifically, ATP-site inhibitors of mTOR are emerging as potential cancer therapeutics [17, 18]. However, a major limitation in the application of these compounds has been the inability to identify patients whose tumors are dependent on aberrant translation control. YB-1 and MTA1 are the first translationally regulated biomarkers to be identified that significantly correlate with disease behavior in prostate cancer. As such, these functional markers may have clinical utility in prospectively stratifying patients who are more or less likely to respond to therapeutics that modulate mRNA translation. Future studies will be required to determine the utility of these approaches for risk stratification and therapy selection. Overall, these findings demonstrate the prognostic power of translationally regulated mRNAs and their protein abundance in forecasting cancer behavior and present a new paradigm for biomarker discovery and development.

## MATERIALS AND METHODS

### Patient population and tissue collection

Prostate tissue samples were taken from a series of 332 men who underwent radical prostatectomy between 1991 and 2003 at the West Los Angeles Veteran's Administration Medical Center (WLA VA) with Institutional Review Board approval. Data including patient age at surgery, race, height, weight, clinical stage, Gleason score, preoperative PSA, surgical specimen pathology (tumor grade, surgical margin status, seminal vesicles invasion, extracapsular extension, and lymph node metastasis), postoperative PSA, and biochemical recurrence status were retrieved from the WLA VA Medical Center database. Patients who received preoperative androgen deprivation or radiation therapy were excluded from this study. Patients were followed for an average of 72.9 months (median = 64 months, range from 1 - 207 months). 56% of the cohort was non-white. The median age of diagnosis was 63 with a mean pre-prostatectomy PSA of 10.2. 43% of the patients had a positive surgical margin. PSA recurrence was defined as a PSA > 0.2, two PSA values at 0.2, or secondary treatment for an elevated post-operative PSA. 129 patients (39%) out of 332 exhibited PSA recurrence (Table 1) and 14 developed metastasis during the course of the study.

### YB-1 and MTA1 genomic DNA mutation analysis in prostate cancer

Using the cbio database (http://www.cbioportal.org/public-portal/) we determined the frequency of genomic mutations or amplification of YB-1 and MTA1 in the Taylor et al. Cancer Cell 2010 [7], Barbieri et al. Nature Genetics 2011 [13], and Grasso et al. Nature 2012 [14] datasets.

### YB-1 and MTA1 mRNA variation study in normal prostate tissue and prostate cancer

The Oncomine (www.oncomine.org) database was used to analyze mRNA levels of YB-1 and MTA1 in normal prostate tissue compared to prostate cancer tissues. The following search terms were utilized for the analysis: gene - *YBX1* or *MTA1*, analysis type (differential analysis) - cancer versus normal analysis, and cancer type - prostate carcinoma. Normal tissues in these datasets denote either normal prostate gland tissues from healthy patients, or normal adjacent prostate gland tissues from prostate cancer patients. Sample annotations were used to define logical groups for comparison (cancer versus normal). Log_2_ median-centered intensity was determined for YB-1 or MTA1 for each set of tissues (cancer or normal) in each study. Using these values, a fold change was determined between normal tissues and cancer tissues. A Student's t-test was performed to generate a P-value. Overall, the YB-1 and MTA1 data represent 1016 unique normal and prostate cancer specimens (Table 2).

### Tissue microarray construction

Tissues were reviewed and selected by two independent pathologists. The tissue microarray (TMA) was constructed using a Manual Tissue Arrayer (MTA, Beecher Instruments Inc.). 0.6 mm diameter coring needles were used to extract representative areas from formalin fixed paraffin embedded surgical tissue blocks. Each tissue was sampled multiple times to include specimens from the primary pathological Gleason grade, the secondary Gleason grade, PIN when available, and adjacent normal regions. At least three cores of each histology type were taken from the surgical blocks and placed into the TMA block. The final TMA was sectioned into 4-micron slices and stained as described below.

### Antibodies and immunofluorescence staining

Immunofluorescence staining was performed as previously described [5]. Samples were stained for YB-1, or co-stained for epithelial marker CK8 and MTA1. Anti-CK8 primary antibody (Covance) was detected with Alexa Fluor 647-conjugated secondary antibody (Life Technologies). Anti-MTA1 (Cell Signaling Technology) and YB-1 (Abcam) primary antibodies were amplified utilizing biotinylated secondary antibody (Vector Labs), and were detected with Alexa Fluor 594-conjugated streptavidin (Life Technologies). Cells were mounted in VECTASHIELD HardSet Mounting Medium with DAPI (Vector Labs). Species-specific isotype controls (Vector Labs) were utilized as negative controls.

### Imaging acquisition and analysis

Isotype control staining in normal and cancerous prostate samples showed negligible background fluorescence in all channels utilized. Imaging for YB-1 expression was performed utilizing the Keyence BZ-9000 BIOREVO fluorescence microscope at 20X magnification. Z-stack images were acquired to accurately sample throughout the depth of the prostate tissue specimens. Whole-cell (cytoplasmic and nuclear) YB-1 expression was quantified utilizing Keyence BZ Image Analysis software. As YB-1 expression is significantly lower in the non-epithelial-derived cells than in epithelial-derived cells in prostate samples, a minimum threshold for YB-1 expression was set to eliminate stromal YB-1 expression from the analysis. Z-stack imaging for MTA1 expression was performed utilizing a Nikon Ti-E fluorescence microscope at 20X magnification with a Coolsnap HQ2 camera and Sutter Lambda LS lamp and shutter. Nuclear MTA1 protein expression levels were quantified in epithelial cells utilizing MATLAB and the MATLAB Image Processing Toolbox (MathWorks). Epithelial cells were identified based on presence of CK8 staining, and nuclear area was identified from DAPI staining.

### Statistical methods

For each patient, the maximum and mean MFI for both YB-1 and MTA1 were calculated for each of the 3 core types (adjacent normal, PIN, and cancer). To compare average intensities across the core classifications a one-way ANOVA was performed. A post-hoc test (Fisher's LSD) was utilized to determine which core types were differentially expressed at the protein level. Spearman correlation coefficients were calculated to assess the relationship between clinical covariates and average intensity levels. Univariate Cox proportional hazards regression models were constructed using standard clinical covariates and markers. The forward stepwise variable selection technique was utilized for the multivariate model. The average mean MFIs within the PIN core subtype were divided into quartiles and plotted using a Kaplan-Meier curve to assess the relationship between marker intensity levels and biochemical recurrence. A log-rank test was used to determine the statistical significance between the quartiles. The nomogram was constructed using the ‘rms’ package in R to visualize and assess the prognostic utility and magnitude of each variable in the multivariate cox model. To assess the prognostic ability of our models the survival C-statistic was computed using the ‘CPE’ package in R. The statistic ranges from 0.5 (not predictive) to 1.0 (perfectly predictive). Statistical analyses was performed with SAS 9.3 (SAS Institute, Cary NC), SPSS 22 (IBM Corp., Armonk, NY), and R Version 3.0.2 (www.R-Project.org, Vienna, Austria). P < 0.05 was considered statistically significant for all analyses.

## SUPPLEMENTARY MATERIAL AND FIGURES


