# Supplementary material for: YB-1 and MTA1 protein levels and not DNA or mRNA alterations predict for prostate cancer recurrence
Source: Oncotarget. 2015 Mar 3;6(10):7470–80. doi: 10.18632/oncotarget.3477 (PMC4480693; doi:10.18632/oncotarget.3477)
Supplement: Supplementary file 1 [file oncotarget-06-7470-s001.pdf]

YB-1 and MTA1 protein levels and not DNA or mRNA alterations predict for prostate cancer recurrence

Supplementary Material

Study	% of tissue composed of tumor
Arredouani et al. CCR 2009	>30%
Grasso et al. Nature 2012	>50%
Holzbeierlein et al. Am J Path 2004	60-80%
LaTulippe et al. Cancer Research 2002	n/a
Liu et al. Cancer Research 2006	90%
Luo et al. Mol Carcinog 2002	100%
Magee et al. Cancer Research 2001	90-100%
Singh et al. Cancer Cell 2002	80%
Taylor et al. Cancer Cell 2010	>70%
Tomlins et al. Nature Genetics 2007	100%*
Vanaja et al. Cancer Research 2003	>80%
Verambally et al. Cancer Cell 2005	n/a
Wallace et al. Cancer Research 2008	n/a
Welsh et al. Cancer Research 2001	50-90%
Yu et al. JCO 2004	>70%

Supplementary Material 1: Percentage of tissue composed of tumor. * This study utilized laser capture microscopy to isolate highly purified prostate tissue/cancer samples (n/a = data not available).

	YB-1		MTA1	
	n	Mean MFI (S.D.)	*n*	Mean MFI (S.D.)
**Adjacent normal**	332	45.2 (14.6)	325	1938 (405)
**PIN**	219	54.2 (18.1)	210	1943 (494)
**Cancer**	332	57.6 (15.9)	329	2050 (469)

Supplementary Material 2: Descriptive statistics for mean MFI in all tissues analyzed. MFI = mean fluorescence intensity.

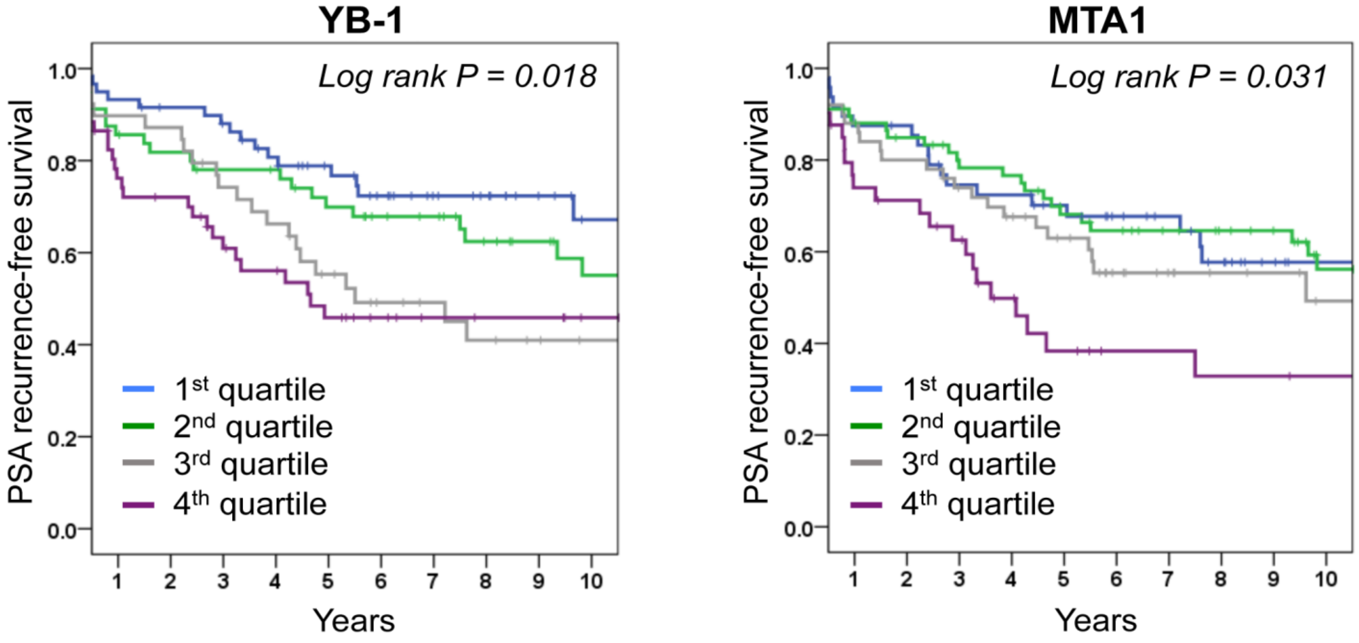

Supplementary Material 3: Kaplan-Meier analysis demonstrates that high protein expression levels of YB-1 or MTA1 within prostatic intraepithelial neoplasia lesions are associated with worse PSA recurrence-free survival.

Quartile Cutoffs		
Quartile	YB-1	MTA1
Q1	<41.6	<1488.5
Q2	41.6-51.1	1488.5-1777.8
Q3	51.1-60.5	1777.8-2031.9
Q4	>60.5	>2031.9

Supplementary Material 4: Quartile cut offs for YB-1 and MTA1.
